# Characterization of the whole plastid genome sequence of *Abies chensiensis* (Pinaceae), an endangered endemic conifer in China

**DOI:** 10.1080/23802359.2018.1521312

**Published:** 2018-10-08

**Authors:** Mi-Li Liu, Ji-Qing Bai, Wan-Lin Dong, Ruo-Nan Wang, Peng-Bin Dong, Ning- Wang, Hong-Yan Liu, Min-Feng Fang

**Affiliations:** a Key Laboratory of Resource Biology and Biotechnology in Western China, Ministry of Education, College of Life Sciences, Northwest University, Xi’an, China;; b College of Pharmacy, Shaanxi University of Chinese Medicine, Xianyang, China

**Keywords:** *Abies chensiensis*, plastid genome, phylogenetic analysis

## Abstract

*Abies chensiensis* Van Tiegh. (Pinaceae) is a rare and endangered endemic conifer in China. In this study, using the Illumina sequencing platform, we firstly characterized its whole plastid genome sequence. Our study revealed that *A. chensiensis* have a typical plastid genome of 121,498 bp in length, comprised of a large single copy region of 76,484 bp, a small single copy region of 42,654 bp and two inverted repeat regions of 1180 bp. A total of 113 genes, 74 protein-coding genes, 35 tRNA, and 4 rRNA genes were identified. The phylogenetic analysis indicated that *A. chensiensis* was placed as a sister to the congeneric *A. sibirica*.

*Abies chensiensis* Van Tiegh. (Pinaceae) is a rare and endangered endemic conifer species in China. Overexploitation, as well as the expansion of human activities and rapid climate change, results in the increasingly declining of *A. chensiensis* wild population resources. It is thus urgent to take effective management measures to conserve this endangered and rare conifer plant. In plant, chloroplast DNA sequence provided valuable phylogenetic signals, owning to its conserved genome characteristics and relatively high evolutionary rates (Wu and Ge 2012). However, some recent studies mainly focused on the reproduction ecology and physiology of *A. chensiensis* (Lai et al. 2003; Zhang et al. 2006). Herein, we first reported the complete plastid genome of *A. chensiensis.*

The fresh needle leaves from a single tree of *A. chensiensis* were collected from Qinling Mountains, Shaanxi Province, China (N34.0062°, E107.8098°). The genomic DNA was extracted from leaf tissues using the modified CTAB method (Doyle and Doyle 1987). DNA sample and voucher specimen (No. ACLZH2015236) of *A. chensiensis* were deposited in the Northwest University Museum (NUM). Then, the fragmented DNAs were subjected to Illumina sample preparation, and pair-read sequencing was conducted on the Illumina Hiseq 2500 platform. In total, about 1,777,826 high-quality reads were obtained and used for the complete plastid genome reference-guided assembly by the MIRA 4.0.2 program (Chevreux et al. 2004) and MITObim v1.7 software (Hahn et al. 2013). Annotation of plastid genome was conducted using the online program Dual Organellar Genome Annotator (DOGMA, Wyman et al. 2004), and then manually adjusted the positions of start codes and stop codes. Eventually, the complete and annotated plastid genome sequence of *A. chensiensis* has been submitted to GenBank with the accession number MH047653. Finally, we drew the circular plastid genome maps using the program OGDRAW (Lohse et al. 2013).

The whole plastid genome of *A. chensiensis* was a typical quadripartite circular molecule with a length of 121,498 bp, which comprises a large single copy (LSC) region of 76,484 bp and a small single copy (SSC) region of 42,654 bp, separated by two inverted repeat regions (IRs) of 1180 bp. The complete plastid genome contains 113 genes, including 74 protein-coding genes, 35 tRNA and 4 rRNA genes. Among them, the genome contained 110 unique genes, 3 genes duplicated in the IRs. A total of 12 genes (*atpF*, *petB*, *petD*, *rpl2*, *rpl16*, *rpoC1*, *trnA-UGC*, *trnG-GCC*, *trnI-GAU*, *trnK-UUU*, *trnL-UAA*, *trnV-UAC*) contained one intron, and two genes (*ycf3*, *rps12*) contained two introns. The overall GC content of *A. chensiensis* plastid genome is 38.3%, while the corresponding values of LSC, SSC, and IR regions are 38.9%, 37.1%, and 37.5%, respectively.

The whole plastid genome sequences of nine species within Pinaceae and two Cupressaceae species were available downloaded from NCBI. All of the twelve plastid sequences were aligned using the software MAFFT (Katoh and Standley 2013) with the default parameters. The phylogenetic analysis was conducted using the program MEGA7 (Kumar et al. 2016) with 1000 bootstrap replicates. The results revealed that *A. chensiensis* was placed as a sister to the congeneric *A. sibirica* with 100 bootstrap value (Figure 1). The plastid genome information reported in this study provided data useful for population genomic and phylogenomic studies of *A. chensiensis*, meanwhile, the characteristics of plastid genome will provide fundamental data for the conservation, utilization, and management of this endangered and rare conifer species.

**Figure 1.**
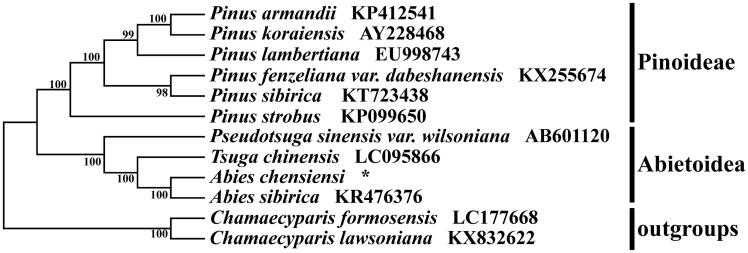
Phylogenetic tree based on twelve complete plastid genome sequences. *The newly generated plastid genome of *Abies chensiensis*.
